# Dynamic subnuclear relocalisation of WRKY40 in response to Abscisic acid in *Arabidopsis thaliana*

**DOI:** 10.1038/srep13369

**Published:** 2015-08-21

**Authors:** Katja Geilen, Maik Böhmer

**Affiliations:** 1 Institut für Biologie und Biotechnologie der Pflanzen, Westfälische Wilhelms-Universität, Münster, Germany

## Abstract

WRKY18, WRKY40 and WRKY60 are members of the WRKY transcription factor family and function as transcriptional regulators in ABA signal transduction in *Arabidopsis thaliana*. Here we show that WRKY18 and WRKY40, but not WRKY60, co-localise with PIF3, PIF4 and PHYB to Phytochrome B-containing nuclear bodies (PNBs). Localisation to the PNBs is phosphorylation-dependent and is inhibited by the general Ser/Thr-kinase inhibitor Staurosporine. Upon ABA treatment, WRKY40 relocalises from PNBs to the nucleoplasm in an OST1-dependent manner. This stimulus-induced relocalisation was not observed in response to other abiotic or biotic stimuli, including NaCl, MeJA or flg22 treatment. Bimolecular fluorescence complementation experiments indicate that while PIF3, PIF4 and PHYB physically interact in these bodies, PHYB, PIF3 and PIF4 do not interact with the two WRKY transcription factors, which may suggest a more general role for these bodies in regulation of transcriptional activity.

The phytohormone Abscisic acid (ABA) regulates plant development and plant adaptation to environmental changes, i.e. seed maturation, seed germination and stomatal aperture regulation in response to drought or pathogens^1^,^2^,^3^. ABA elicits fast physiological responses, like the activation of ion channels in response to drought, but also induces long term responses by modulation of gene expression. Gene expression in response to ABA was extensively studied in a number of different cell types and model systems^4^,^5^,^6^. Regulation of transcriptional activity in response to ABA is transmitted via a core signalling cascade. ABA is perceived by receptors of the PYRABACTIN RESISTANCE 1-LIKE/ REGULATORY COMPONENT OF ABA RECEPTOR (PYL/RCAR) protein family^7^,^8^. The RCAR proteins then interact with PROTEIN PHOSPHATASE 2C (PP2C) isoforms, negative regulators of ABA signalling, and thereby release inhibition of SUCROSE NONFERMENTING 1-RELATED PROTEIN KINASE 2 (SNRK2) isoforms^8^,^9^,^10^,^11^. SNRK2 kinases activate reactive oxygen species (ROS) production by phosphorylation of NADPH oxidase RbohF^12^,^13^. Using gene chip analyses, we have recently shown that regulation of gene expression is in part transmitted via the production of REACTIVE OXYGEN SPECIES (ROS)^14^. In addition, SNRK2 kinases directly phosphorylate and activate transcription factors of the ABSCISIC ACID RESPONSIVE ELEMENTS-BINDING FACTOR family (AREB/ABF)^15^,^16^,^17^,^18^.

Besides ABF proteins, other transcription factor families play a role in ABA signalling, including MYB DOMAIN PROTEINS, ETHYLENE RESPONSE FACTORS, NUCLEAR FACTOR Y SUBUNIT A5 and WRKY DNA-BINDING PROTEINS (WRKY)^1^,^19^,^20^,^21^,^22^,^23^,^24^. Two WRKY proteins from *Avena fatua* (common wild oat), *Af*WRKY1 and *Af*WRKY2, which are orthologues of *A. thaliana* WRKY40, were among the first identified WRKY transcription factors. They regulate gene expression during seed germination^25^. In Arabidopsis, a number of Arabidopsis WRKY transcription factors have been identified to function in ABA signalling, including WRKY2, WRKY18, WRKY40, WRKY46, WRKY60 and WRKY63^26^,^27^,^28^,^29^,^30^. WRKY18, WRKY40 and WRKY60 belong to the IIa subgroup of WRKY transcription factors. WRKY18 and WRKY40 play a role in plant defence^31^,^32^,^33^,^34^,^35^,^36^,^37^, and similarly to *Af*WRKY1 and *Af*WRKY2, WRKY40 also negatively regulates ABA responses during seed germination and postgerminative growth^28^,^29^. A number of ABA-regulated genes, including RCAR receptors, ABA INSENSITIVE 1 (ABI1), ABI2 and ABF transcription factors of Arabidopsis are differentially expressed in the *wrky40* mutant and are in part directly transcriptionally regulated by WRKY40^28^,^38^. WRKY40 therefore constitutes a prerequisite for functional ABA signalling by facilitating expression of ABA-signalling components. For WRKY18 and WRKY60, contrasting findings suggested that they both act either as negative regulators^28^ or as positive regulators of ABA signalling^29^. It was suggested that in response to ABA, these WRKY transcription factors translocate from nucleus to cytoplasm and interact with the H SUBUNIT OF MG-CHELATASE (GUN5), a putative ABA receptor protein^28^.

Sub-compartmental relocalisation has been described for other ABA responsive proteins, including ABA-ACTIVATED PROTEIN KINASE INTERACTING PROTEIN (AKIP1) and UBP1 INTERACTING PROTEIN 2a (UBA2a)^39^,^40^,^41^. In response to ABA, these proteins relocalise from the nucleoplasm to nuclear bodies^39^,^40^,^41^. Spatial and temporal organisation of the nucleus is a key requirement for regulation of splicing, transcription and thereby regulation of the transcriptome^42^. The nucleus of plants and animals consists of various subnuclear compartments that have different cellular functions^43^. The relationship between subnuclear localisation and signalling is not very well understood in plants. One well studied example, however, is the relocalisation of Phytochrome B (PHYB), a central light-dependent regulator in plants. PHYB is synthesized in the cytosol in its inactive P_r_ form. Upon excitation by red light it is converted into the active P_fr_ form that relocalises to PHYB-containing nuclear bodies (PNBs)^44^,^45^,^46^,^47^,^48^,^49^,^50^. Inside PNBs, PHYB co-localises with PHYTOCHROME INTERACTION FACTOR 3 (PIF3), PIF7 and CONSTITUTIVE PHOTOMORPHOGENIC 1 (COP1)^51^.

Whether subnuclear relocalisation is a regulatory mechanism for ABA-regulated transcription factors has not been systematically studied. Here we show subnuclear relocalisation of WRKY40 transcription factors in response to ABA in an OPEN STOMATA 1 (OST1)-dependent manner. Subnuclear relocalisation might constitute a new regulatory mechanism, how ABA modulates transcription factor activity.

## Results

### Subnuclear localisation of WRKY18, WRKY40 and WRKY60

**Figure 1:**
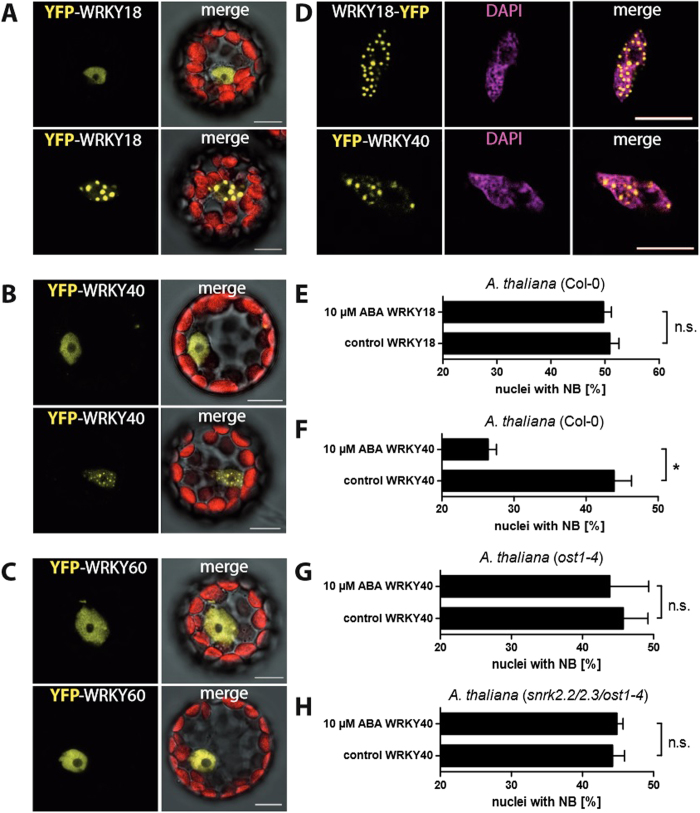
Subnuclear localisation of WRKY18, WRKY40 and WRKY60. Confocal images of *A. thaliana* protoplast (Col-0) transformed with either YFP-WRKY18 (**A**) or YFP-WRKY40 (**B**) or YFP-WRKY60 (**C**) (scale bar 10 μm). (**D**) DAPI staining of nuclei expressing WRKY18-YFP and YFP-WRKY40 in transiently transformed *A. thaliana* seedlings (scale bar 10 μm). Bar charts show changes in subnuclear localisation in response to ABA for WRKY18 from nuclear bodies to nucleoplasm in Col-0 (**E**) and for WRKY40 in Col-0 (**F**), *ost1*–*4* mutant (**G**) and *snrk2.2/2.3/ost1*–*4* mutant (**H**) protoplasts (means ± SE). Statistical analysis was performed with Fisher Exact test (P < 0.05). Asterisks indicate significant difference when significance was obtained in at least 3 biological replicates.

The subnuclear localisation of three WRKY transcription factors with a known role in ABA signal transduction, WRKY18, WRKY40 and WRKY60, was analysed 15 hours after transformation of *A. thaliana* wildtype (Col-0) protoplasts. WRKY60 localised exclusively to the nucleoplasm. WRKY18 and WRKY40 localised to the nucleoplasm or to nuclear bodies (Fig. 1A–C). Size and number of nuclear bodies was different for each WRKY transcription factor, with larger nuclear bodies present for WRKY18. Identical localisation to nuclear bodies was also identified after transient expression of WRKY18 and WRKY40 in Arabidopsis seedlings (Fig. 1D). DAPI staining confirmed the nuclear body localisation of WRKY18 and WRKY40 within the nuclei (Fig. 1D). No co-localisation of nuclear bodies with chromatin could be detected. After transient expression in *N. benthamiana,* nuclear bodies could be seen from the onset of protein expression and did not result from elevated protein accumulation (Figure S1). Likewise, Propidium iodide (PI) cell death staining of Arabidopsis protoplasts showed that no cell death occurred in nuclei where WRKYs were localised to nuclear bodies (Figure S2). For a quantitative analysis, the percentage of nuclei with WRKYs localised to nuclear bodies was calculated after transient protoplast transformation. WRKY18 and WRKY40 localised to nuclear bodies in 51% or 44% of nuclei, respectively (Fig. 1E,F).

### Increased nucleoplasmic localisation of WRKY40 in response to ABA

To test the effect of ABA on WRKY localisation, transformed *A. thaliana* protoplasts (Col-0) were incubated with 10 μM ABA for 10–30 min and subnuclear localisation was quantified (Fig. 1E,F). For WRKY18 the percentage of nuclei with nuclear body localisation did not significantly change. For WRKY40, however, the percentage of nuclei with nuclear body localisation decreased from 44% to 26%.

To investigate the involvement of the ABA core signalling pathway in ABA-dependent localisation of WRKY40, localisation experiments were performed with protoplasts of the Arabidopsis SNRK2.6 kinase mutant *ost1*–*4* and of the *snrk2.2/2.3/ost1*–*4* triple mutant. No changes in localisation were observed for WRKY40 in response to ABA in *ost1*–*4* and *snrk2.2/2.3/ost1*–*4* mutant protoplasts (Fig. 1G,H), indicating requirement for the ABA core signalling pathway.

**Figure 2:**
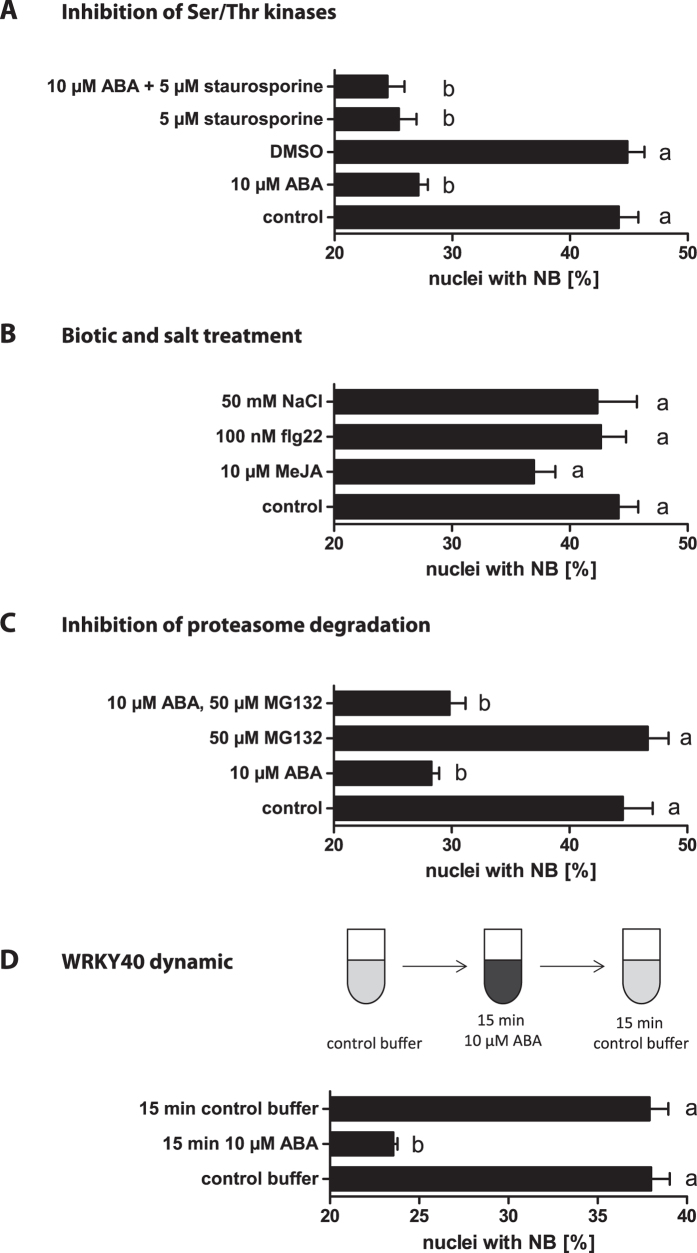
Quantification of WRKY40 nuclear bodies after several treatments. Changes in WRKY40 subnuclear localisation in *A. thaliana* protoplasts Col-0 in response to the Ser/Thr kinase inhibitor Staurosporine (**A**), in response to NaCl, MeJA and flg22 (**B**) and in response to the proteasome inhibitor MG132 (**C**). WRKY40 subcellular localisation dynamics after 15 minutes of ABA treatment, followed by a buffer exchange with control buffer (**D**). Statistical analysis was performed with Fisher Exact test (P < 0.05). Letters indicate significance groups with significant differences observed in at least 3 biological replicates.

A more nucleoplasmic localisation of WRKY40, comparable to ABA-treated protoplasts, was also observed when *A. thaliana* (Col-0) protoplasts were treated with the general Ser/Thr-kinase inhibitor Staurosporine (Fig. 2A). Localisation to nuclear bodies was reduced from 44% to 25%. This indicates that the subnuclear localisation of WRKY40 to nuclear bodies contains a phosphorylation-dependent component. Combined ABA and Staurosporine treatment did not further enhance nucleoplasmic localisation.

In order to determine the specificity of ABA in WRKY localisation, elevated NaCl treatment was tested as another abiotic stress stimulus. No localisation changes in response to NaCl were observed in *A. thaliana* (Col-0) protoplasts (Fig. 2B). Since WRKY40 and the closely related transcription factors WRKY18 and WRKY60 were previously shown to be involved in immune signalling, flg22, a pathogen elicitor, and MeJA, as a phytohormone analogue of biotic signalling were tested^32^,^33^,^34^,^35^,^36^,^37^. In response to MeJA and flg22 treatment, no significant changes in WRKY40 localisation were detectable, indicating that increased WRKY40 nucleoplasmic localisation is stimulus-specific.

Taken together, these data indicate that WRKY40 has an increased nucleoplasmic localisation specifically in response to ABA. WRKY40 localisation depends on phosphorylation and a functional ABA core signalling pathway.

### ABA dependent WRKY40 relocalisation is highly dynamic

To test whether the localisation changes in response to ABA are a result of a dynamic relocalisation of WRKY40 or the outcome of degradation in the nuclear bodies and new protein synthesis, *A. thaliana* protoplasts were treated with the proteasome inhibitor MG132. MG132 itself had no effect on WRKY40 localisation and in combination with ABA did not affect the increased nucleoplasmic localisation (Fig. 2C).

In order to further analyse the localisation dynamics of WRKY40, protoplasts were treated with ABA for 15 minutes and then ABA was removed by a buffer exchange. The percentage of nuclei with nuclear body localisation decreased after 15 min ABA treatment and recovered after washing and 15 min incubation in control buffer (Fig. 2D).

In conclusion, the ABA dependent increase in nucleoplasmic localisation of WRKY40 is the result of a highly dynamic process where WRKY40 protein shuttles between nuclear bodies and nucleoplasm.

### WRKY18 and WRKY40 localise to Phytochrome B-containing nuclear bodies (PNBs)

**Figure 3:**
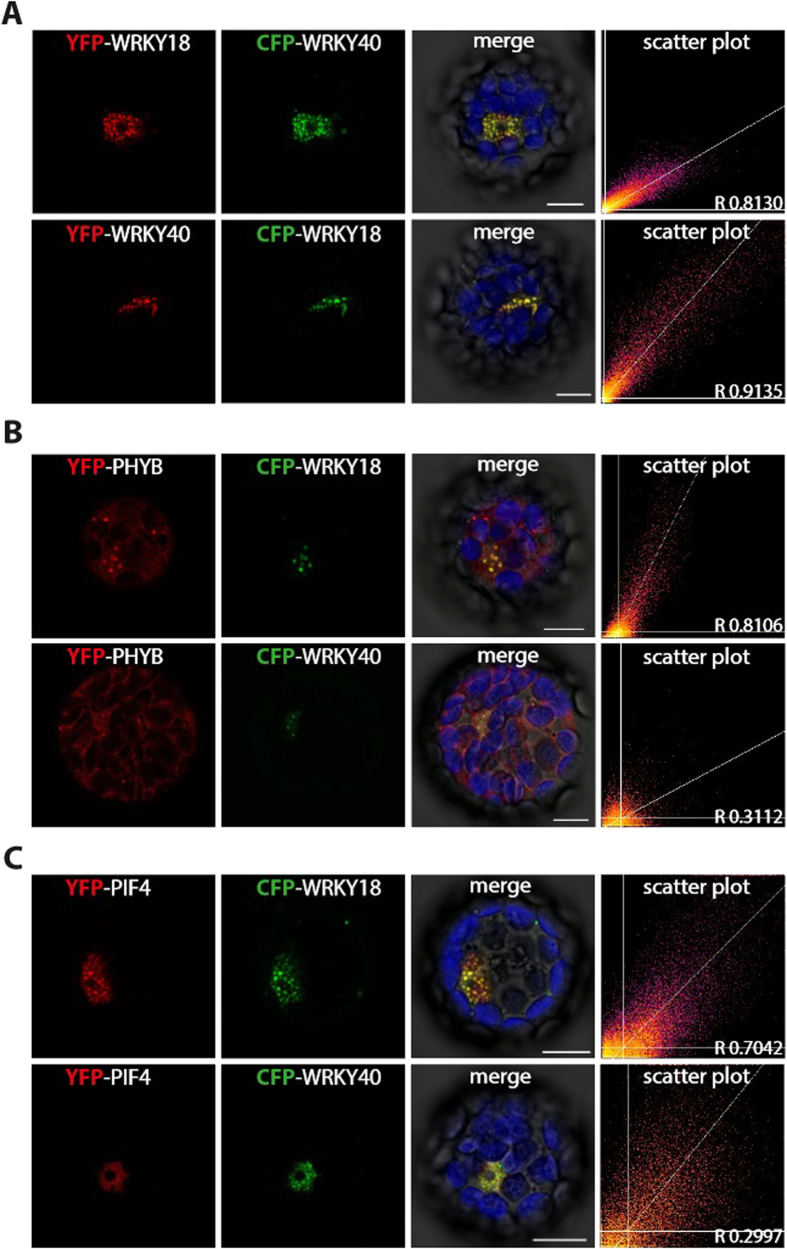
Co-localisation studies in *A. thaliana* Col-0 protoplasts. Confocal images and scatter plots of either co-localisation WRKY18 with WRKY40 (**A**) or WRKY18 and WRKY40 with Phytochrome B (**B**) or WRKY18 and WRKY40 with PIF4 (**C**) (scale bar 10 μm).

In order to investigate the identity of the nuclear bodies, co-localisation experiments were performed. WRKY18 and WRKY40 localised to identical nuclear bodies in Arabidopsis wildtype protoplasts (Fig. 3A). Further co-localisation experiments with marker proteins, including splicing factors and phytochromes, identified PHYB, PIF3 and PIF4 as proteins co-localising to the same nuclear bodies. WRKY18 nuclear body localisation did strongly overlap with PIF3, PIF4 and PHYB localisation in *A. thaliana* protoplasts (Fig. 3B,C). WRKY40 nuclear body localisation, however, did only overlap with a subset of PIF3-, PIF4- and PHYB-containing nuclear bodies (Fig. 3B,C).

**Figure 4:**
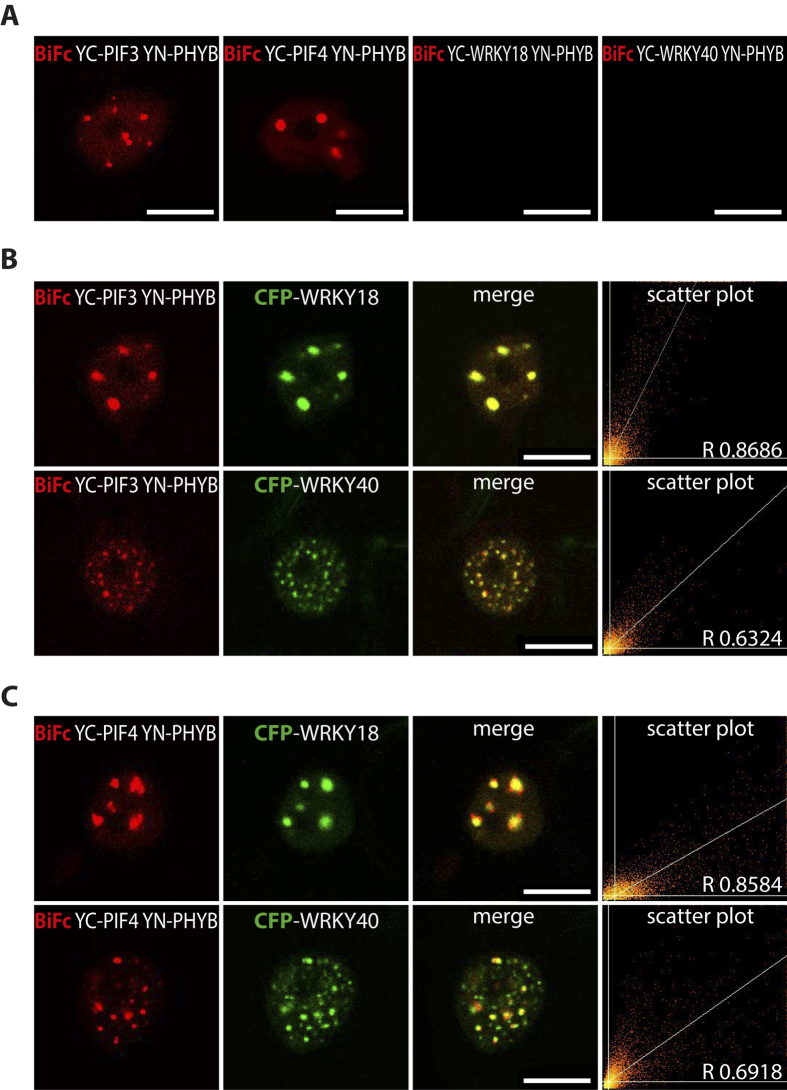
BiFc and co-localisation studies in transient transformed *N. benthamiana* leaves. Bimolecular fluorescence complementation experiments of YN-PHYB and YC-PIF3, YC-PIF4, YC-WRKY18 and YC-WRKY40 (**A**). Confocal images and scatter plots of either co-localisation of WRKY18 and WRKY40 with BiFc of YN-PHYB and YC-PIF3 (**B**) or co-localisation of WRKY18 and WRKY40 with BiFc of YN-PHYB and YC-PIF4 (**C**) (scale bar 10 μm).

To test for physical interactions between PIF3, PIF4, PHYB and WRKY proteins, bimolecular fluorescence complementation (BiFC) experiments were performed in *N. benthamiana*. Known interactions between PIF3, PIF4 and PHYB^52^,^53^ served as control. WRKY18 and WRKY40 did not interact with PIF or PHYB proteins (Fig. 4A). Co-localisation and combined co-localisation and BiFC experiments with YC-PIF3/YC-PIF4, YN-PHYB and CFP-WRKY18/CFP-WRKY40 however confirmed co-localisation to the same nuclear bodies (Fig. 4B,C and Figure S3).

## Discussion

WRKY transcription factors are regulated via transcriptional regulation, homo- and heterodimerisation, phosphorylation and epigenetic control^54^. WRKY18, WRKY40 and WRKY60 function as transcriptional regulators in ABA signal transduction. We studied their potential regulation via subnuclear relocalisation in response to ABA. All three WRKY transcription factors constitutively localise to the nucleus. While WRKY60 primarily localises to the nucleoplasm, WRKY18 and WRKY40 localise to the nucleoplasm and to nuclear bodies. Co-localisation experiments with PHYB and PIF proteins identified the nuclear bodies as PNBs. WRKY18 localised to larger nuclear bodies, reminiscent of PHYB localisation when more PHYB is in the P_fr_ form. WRKY40 contrastingly localised to smaller bodies, similar to when more PHYB remains in the P_r_ form^48^. In a small number of experiments, transformation of *A. thaliana* protoplasts also led to a dotted cytoplasmic localisation of WRKY40. In contrast to previous reports, however, cytoplasmic localisation in our experiments was independent of ABA treatment and varied between individual experiments^28^.

Although WRKY18, WRKY40 and WRKY60 are phylogenetically closely related, they do not share their subnuclear localisation. The WRKY transcription factor *Hv*WRKY2, a homologue of *At*WRKY40 in barley, however, was shown to also localise to nuclear bodies in transiently transformed barley leaves^55^. This suggests that the localisation of individual WRKYs to PNBs is conserved in dicotyledonous and monocotyledonous plants.

Under control conditions, WRKY40 localises to nuclear bodies in about 50% of nuclei in *A. thaliana* protoplasts. Upon treatment with ABA, WRKY40 responds by subnuclear relocalisation to the nucleoplasm in *A. thaliana* wildtype protoplasts. However, neither in *A. thaliana snrk2.2/2.3/ost1*–*4* mutant protoplasts nor in *A. thaliana ost1*–*4* mutant protoplasts does WRKY40 relocalise to the nucleoplasm in response to ABA, suggesting a dependency on ABA core signalling components. The protoplasts on our assays derive primarily from mesophyll cells. Based on cell type specific expression data, SnRK2.2 and SnRK2.3 are more strongly expressed than SnRK2.6 in mesophyll cells^56^. Nevertheless SnRK2.6 seems to play a prominent role in WRKY40 relocalisation with no added effect in the *snrk2.2/2.3/ost1*–*4* triple mutant. Whether SnRK2.6 is directly activated during the relocalisation event or whether SnRK2.6 expression is a prerequisite, leading e.g. to differential gene expression in these plants, remains to be tested.

Treatment with the general Ser/Thr-kinase inhibitor Staurosporine also reduces WRKY PNB-localisation to an extent similar to ABA treatment. Co-treatment with ABA and Staurosporine did not show an additive effect. This indicates that the initial localisation to nuclear bodies and not the relocalisation to the nucleoplasm is phosphorylation dependent.

Two models may explain the dynamics of WRKY relocalisation. Either WRKY proteins shuttle between PNBs and the nucleoplasm, or WRKY proteins are constantly degraded in PNBs and newly synthesised, upon which they are prevented from entering PNBs by the action of ABA or Staurosporine. Since combined treatment with the proteasome inhibitor MG132 and ABA is able to induce WRKY40 relocalisation, WRKY40 relocalisation is not mediated by proteasome degradation. This is further supported by reversible localisation to nuclear bodies after removal of ABA by buffer exchange for 15 minutes. This is also in accordance with similar observations for PHYB, where upon photobleaching of PNBs, these were readily refilled with fluorescently labelled PHYB protein, indicating a dynamic exchange between PNBs and the nucleoplasm^57^.

The function of PNBs during light signalling is still controversial^51^. A widely proposed model is that PNBs are sites of degradation for individual transcription factors, e.g. PIF3. A similar model was suggested for ABA INSENSITIVE 5 (ABI5), a positive regulator of ABA-inhibition of seed germination. ABI5 localises to nuclear bodies when expressed with ABA-INSENSITIVE FIVE BINDING PROTEIN (ABF), potentially leading to ABI5 degradation^58^. Our proteasome inhibitor experiments indicate, however, that WRKY40 is not degraded in PNBs. Also there is no indication that ABI5 and WRKY40 are localised to the same nuclear bodies.

In a second model for PNB function, it was suggested that transcription factors bring their target genes to the vicinity of PNBs and where they regulate target gene expression^51^. A comparable model was suggested in ABA signalling. The RNA-binding protein UBA2a and its *Vicia faba* homologue AKIP1, both localise to nuclear bodies in response to ABA^39^,^40^,^41^. Nuclear body localisation of AKIP1 was shown to be dependent on phosphorylation by the OST1-homologue AAPK and transcription of AKIP1 target genes. Phosphorylated AKIP1, bound to its target transcripts, is supposed to shuttle to nuclear bodies^39^,^40^.

PNBs were also suggested to serve as storage depots for active transcription factors^51^. LIGHT HARVESTING CHLOROPHYLL A/B BINDING (LHCB) proteins are positive regulators of ABA signalling. Downregulation of any of the six LHCB proteins results in ABA-insensitivity in seed germination. ABA enhances LHCB expression, while WRKY40 represses their expression by directly binding to the promoter of LHCB genes^59^. ABA-dependent subnuclear compartmentalisation might explain how ABA fine-tunes WRKY40 activity on target genes, such as LHCBs, by sequestering a subset of WRKY40 protein into inactive subnuclear compartments. PNB localisation may also attribute to WRKY dimerisation. In gel-shift assays WRKY18/WRKY40 heterodimers bind more strongly to w-box containing sequences than the respective homodimers. WRKY40/WRKY60 heterodimers, on the other hand, show a decrease in binding activity^35^. Higher order complexes of all three WRKY proteins often abolished promoter binding^38^.

To clarify the biological function of ABA-induced WRKY40 relocalisation from PNBs to nucleoplasm, and to determine the subnuclear location where WRKY40 is active, transcriptional activity of target gene-promotors would need to be quantified in protoplasts with nuclear body and nucleoplasm localisations of WRKY 40 using fluorescent reporters.

Many developmental processes, e.g. seed germination, require to adopt growth to the environmental situation, including light and abiotic stresses. ABA- and light signalling interact at multiple hubs to allow seed germination and development under various light and stress conditions. PIF1 represses seed germination by regulating the expression of ABA-biosynthesis genes^60^. In a *pif3* mutant background, WRKY18 and WRKY40 were upregulated after 1h light irradiation^61^. Furthermore, a number of transcription factors of light and ABA signalling, including FHY3/FAR1, HY5, ABI5, ABI4 and WRKY40 bind to the promotor of ABI5^62^. ABI5 is a central signalling hub in ABA and light signalling. Overexpression of ABI5 for example led to shorter hypocotyls under FR and R conditions^63^. WRKY40 could have a possible role during light and ABA signalling at the level of ABI5 regulation. Possibly pre-formation of PNBs under light is needed for WRKY40 localisation to PNBs. Further studies will be necessary to link WRKY relocalisation to physiological processes in plants and to identify additional stimuli that lead to WRKY relocalisation.

## Methods

### Plant material and growth conditions

*Arabidopsis thaliana* ecotype Columbia (Col-0) was used for all localisation experiments. Arabidopsis mutant lines *ost1*–*4* (SALK_008068) and *snrk2.2/2.3/ost1*–*4* (GABI-Kat 807G04, Salk_107315, SALK_008068) were described previously^17^,^64^. *A. thaliana* plants were grown for 4–5 weeks in a plant growth chamber at 23 °C in 12 h days with 50–75 μEm^−2^ s^−1^ illumination and 50% relative humidity. *N. benthamiana* plants were grown in soil in the greenhouse at 22 °C in 14 h days with 270 μEm^−2^ s^−1^ and 60% relative humidity. 25 days old *N. benthamiana* plants were used for Agrobacteria mediated transient expression.

### Cloning of WRKY18, WRKY40, WRKY60, PHYB, PIF3, PIF4

Full length cDNAs corresponding to WRKY18 (At4g31800), WRKY40 (At1g80840) and WRKY60 (At2g25000) were amplified by PCR using the primer pairs WRKY18_f, WRKY18_r and WRKY18_r2, WRKY40_f and WRKY40_r, WRKY60_f and WRKY60_r (Table S1). Amplification products were cloned into pDONR221 (Invitrogen) by BP-reaction and subcloned into the destination vectors pXNSG-YFP, pXCSG-YFP^65^, pXNSG-CFP, pSYN and pSYC^66^. pGPTVII-BAR-35S-mVenus-PIF3 and PIF4; pGPTVII-HYG-35S-SPYNE-PIF3; PIF4 and PHYB, pGPTVII-KAN-35S-SPYCE-M-PIF3 and PIF4 were provided by Jörg Kudla. pXNSG-XFP vectors were provided by Jane Parker. pDONR207-PHYB was provided by Dierk Wanke and was cloned into pXNSG-YFP.

### Transient expression in *Nicotiana benthamiana* and *Arabidopsis thaliana*

Overnight cultures of Agrobacteria strain GV3101:pmp90RK, containing binary vectors and strain GV3101:pmp90, containing the silencing inhibitor p19 were combined, diluted to OD_600_ 0.8 in infiltration media (10 mM MgCl_2_; 10 mM MES pH 5.6; 100 μM Acetosyringon) and incubated at RT for two hours. Two leaves per tobacco plant were infiltrated and grown under continuous light for two days. For transient expression in *A. thaliana* cotyledons, an existing transient expression protocol was optimized for the Agrobacteria strain GV3101:pmp90RK^67^. 10 ml Agrobacteria solution was prepared as described for tobacco infiltration, supplemented with 400 μM Acetosyringone. 4-day-old seedlings grown on 0.5 MS Agar plates were submerged with 10 ml Agrobacteria solution and placed in a desiccator for vacuum infiltration. Infiltrated seedlings were incubated for 2 days in a plant growth chamber. Protoplast transformation was performed as previously described^68^.

For quantification of nuclear body-containing nuclei, at least 100 cells were counted in a minimum of three independent biological experiments. 10 μM ABA in 0.1% ethanol for *N. benthamiana* or in 3 mM MES for *A. thaliana*, 5 μM staurosporine in 2% DMSO, 50 mM sodium chloride in water, 100 nm flg22 in water, 10 μM MeJA in water, 1 μg/ml DAPI in water, 50 μM MG132 and 5 μg/ml Propidium iodide (PI) in water were directly infiltrated into *N. benthamiana* leaves or directly added to protoplast solution. Scatter plot analysis of co-localisation within the nucleus was performed with the Image Processing and Analyse in Java (Image J 1.47v) co-localisation threshold plug in by drawing a region of interest (ROI) around the nucleus.

### Confocal microscopy

Confocal microscopy was performed using an inverted Leica DMIRE2 microscope equipped with a Leica TCS SP2 laser scanning device. Fluorescence was detected as described: YFP/mVenus/BiFc—excitation at 514 nm (Ar/Kr laser), scanning at 530–600 nm; CFP—excitation at 458 nm (Ar/Kr laser), scanning at 465–510 nm; DAPI—excitation with UV-laser, scanning at 440–480 nm; PI—excitation at 488 nm (Ar/Kr laser), scanning at 610–650 nm. All images were acquired using a 63x/1.20 water-immersion objective (HCX PL Apo CS) from Leica.

## Additional Information

**How to cite this article**: Geilen, K. and Böhmer, M. Dynamic subnuclear relocalisation of WRKY40 in response to Abscisic acid in *Arabidopsis thaliana*. *Sci. Rep.* **5**, 13369; doi: 10.1038/srep13369 (2015).

## Supplementary Material

Supplementary Information
