# Supplementary material for: Dynamic subnuclear relocalisation of WRKY40 in response to Abscisic acid in Arabidopsis thaliana
Source: Sci Rep. 2015 Aug 21;5:13369. doi: 10.1038/srep13369 (PMC4642543; doi:10.1038/srep13369)
Supplement: Supplementary Information [file srep13369-s1.pdf]

Dynamic subnuclear relocalisation of WRKY40 in response to Abscisic acid in *Arabidopsis thaliana*

Katja Geilen, Maik Böhmer*

Short title: ABA dependent WRKY relocalisation

Institut für Biologie und Biotechnologie der Pflanzen, Westfälische Wilhelms-Universität, Münster, Germany

*Corresponding author. Present address: Westfälische Wilhelms-Universität, IBBP, Schlossplatz 7, 48149 Münster, Germany. E-mail: m.boehmer@uni-muenster.de

Supporting Information Legends

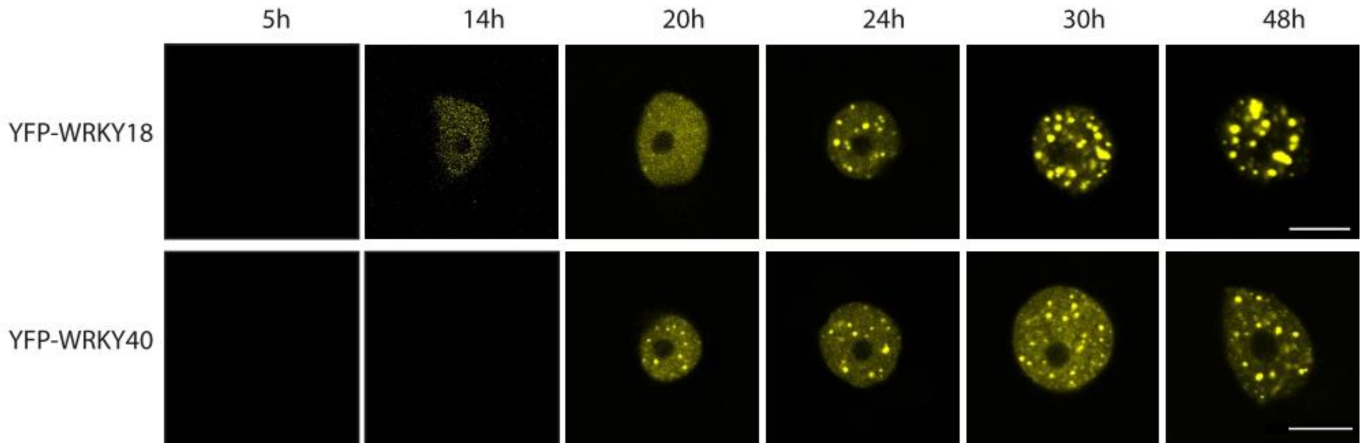

Figure S 1 Expression time course of YFP-WRKY18 and YFP-WRKY40. Confocal images of YFP-WRKY18 and YFP-WRKY40 in transiently transformed *N. benthamiana* leaves after different time points (scale bar 10 µm).

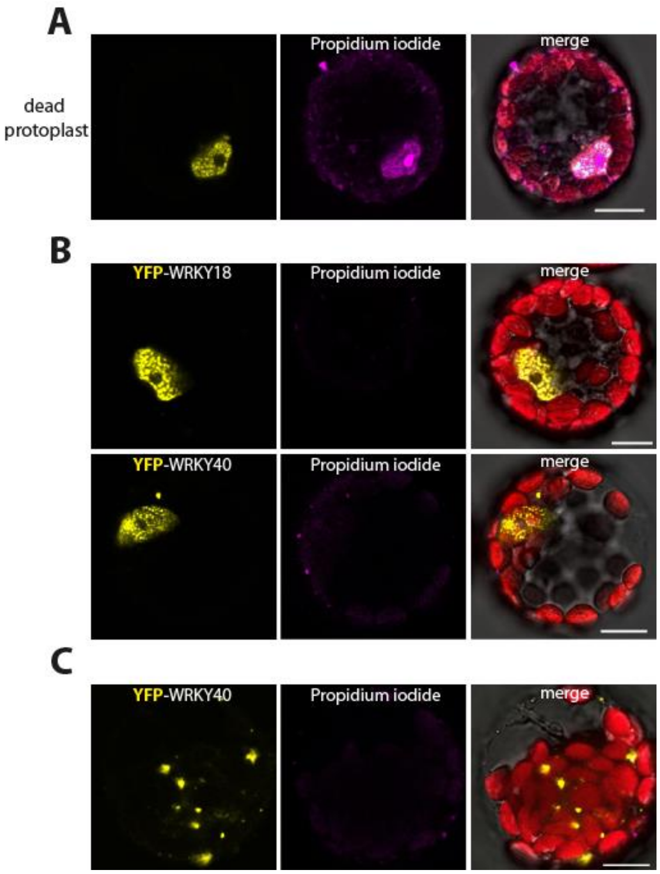

Figure S 2 Viability test of *A. thaliana* protoplasts. Confocal images of Propidium iodide staining (5 µg/ml) of *A. thaliana* protoplasts for identification of dead cells (A) and protoplasts expressing either YFP-WRKY18 or YFP-WRKY40 in nuclear bodies (B) as well as expressing YFP-WRKY40 in cytoplasm (C) (scale bar 10 µm).

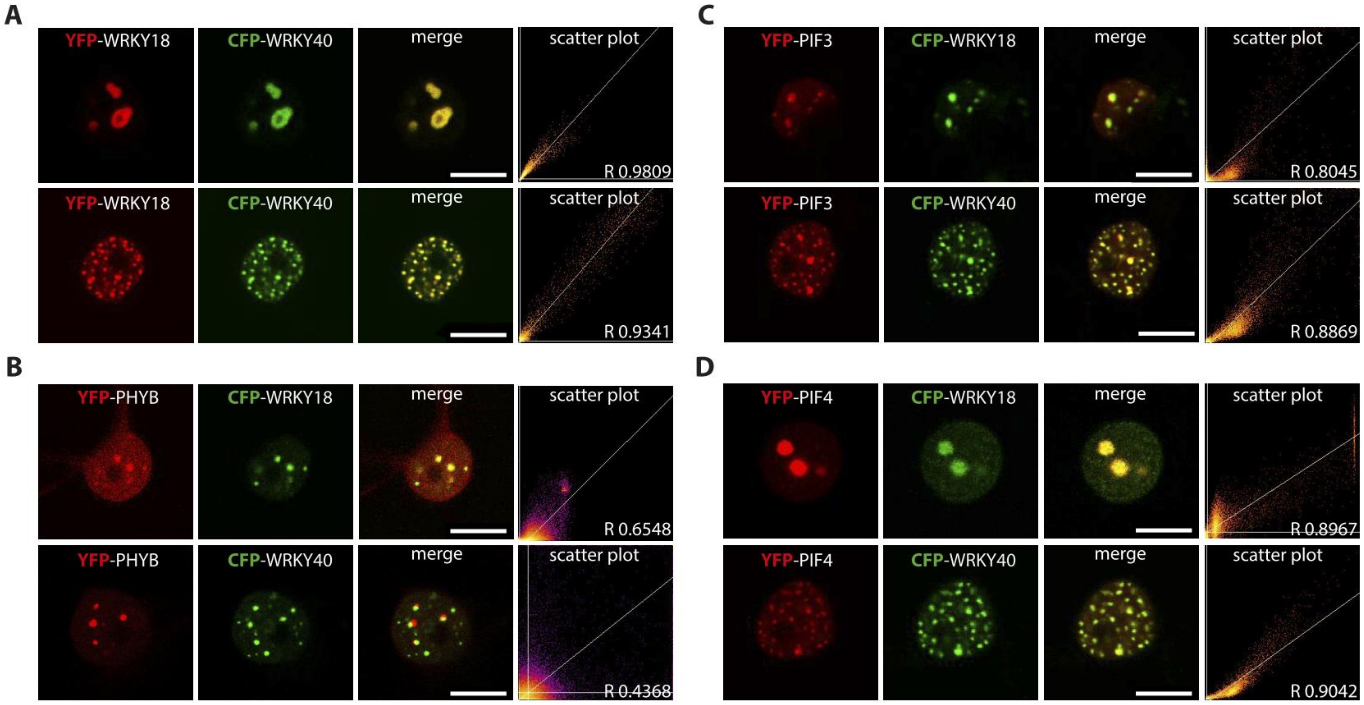

Figure S 3 Co-localisation studies in *N. benthamiana* leaves. Confocal images and scatter plots of either co-localisation of WRKY18 with WRKY40 (A) or WRKY18 and WRKY40 with Phytochrome B (B) or WRKY18 and WRKY40 with PIF3 (C) or WRKY18 and WRKY40 with PIF4 (D) (scale bar 10 µm).

Tabelle S 1 Oligonucleotide sequences for cloning of WRKY18, WRKY40 and WRYK60 into pDONR201.

WRKY18_f	GGGGACAAGTTTGTACAAAAAAGCAGGCTTAATGGACGGTTCTTCGTTT
WRKY18_r	GGGGACAAGTTTGTACAAAAAAGCTGGGTATCATGTTCTAGATTGCTC
WRKY18_r2	GGGGACCACTTTGTACAAGAAAGCTGGGTATGTTCTAGATTGCTCCAT
WRKY40_f	GGGGACAAGTTTGTACAAAAAAGCAGGCTTAATGGATCAGTACTCATCC
WRKY40_r	GGGGACAAGTTTGTACAAAAAAGCTGGGTACTATTTCTCGGTATGATT
WRKY60_f	GGGGACAAGTTTGTACAAAAAAGCAGGCTTAATGGACTATGATCCCAAC
WRKY60_r	GGGGACAAGTTTGTACAAAAAAGCTGGGTATCATGTTCTTGAATGCTCTA
